# Major Stressors among Korean Adolescents According to Gender, Educational Level, Residential Area, and Socioeconomic Status

**DOI:** 10.3390/ijerph15102080

**Published:** 2018-09-21

**Authors:** Subin Park, Hyesue Jang, Eun-Sun Lee

**Affiliations:** 1 Department of Research Planning, Mental Health Research Institute, National Center for Mental Health, Seoul 04933, Korea; hsjang0315@gmail.com; 2 Department of Psychology, Korea University, Seoul 02841, Korea; chachagrace918@gmail.com

**Keywords:** adolescent, stress, South Korea

## Abstract

Adolescents are exposed to many stressors which have been associated with poor mental health. Using data from the 2015 Korea Youth Risk Behavior Web-based Survey, we identified the major stressors among Korean adolescents based on gender, current educational level, residential area, and socioeconomic status (SES). The major stressors among girls were relationship- and appraisal-related factors, whereas boys more often reported health- and conflict-related factors. High school students more often reported academic performance and family circumstances as major stressors, whereas middle school students tended to report conflict-related factors. Urban adolescents reported academic performance and conflicts with parents as major stressors while rural adolescents reported conflicts with teachers and peer relationship problems. Finally, adolescents of lower SES reported multiple factors, including relational and family problems, as major stressors; contrarily, among those of higher SES, the primary stressor was uniquely related to academic performance. This result is significant in that adolescents’ stress levels, as well as the types of major stressors, vary depending on individual factors. It could also be beneficial for developing and implementing individualized and thus more efficient stress-management strategies.

## 1. Introduction

Adolescence is a period of transition, one that entails rapid, novel, and unexpected changes in the physical, social, emotional, and cognitive domains [1]. Massive physical and psychological changes, including drastic growth, sexual maturation, increased importance and demands of social relationships, as well as a heightened desire for independence, occur simultaneously, characterizing the period as full of “storm and stress” [2]. These developmental challenges can be highly overwhelming and stressful for adolescents [3]. In addition, it has been noted that concomitant neurobiological changes could also be responsible for the increased vulnerability to stress. According to empirical studies, disproportionate development between the subcortical area and prefrontal cortex, appears to be more pronounced during adolescence than during childhood or adulthood [2]. To be specific, the relatively protracted developmental timeframe of the prefrontal cortex compared to the subcortical structures (e.g., amygdala) can make adolescents more vulnerable to poor emotion control and thus expose them to more perceived stress and anxiety. Furthermore, considering that coping strategies mature and become diversified with age, adolescents, especially younger ones, are not as likely to have achieved and used adaptive coping strategies. As a result, significant changes and stressors in adolescence may affect adaptive processes [4].

Stress can have detrimental effects on adolescents’ psychosocial adjustment not only in the short term through exposure to acute and temporal stressors but also in the long term by a considerably long exposure to ongoing stressors [5,6]. As a result, an increasing number of children and adolescents have been reporting psychosomatic symptoms (e.g., pain, fatigue and tension) in response to perceived stress [7]. In addition, higher levels of stress are related to greater emotional complaints (e.g., negative affect such as irritability and feeling nervous or sad), and low self-esteem [8]. As a result, increased stress has been indicated as a key risk factor for poor mental health, such as depression, suicide attempts and anxiety problems in adolescents [9,10,11]. Moreover, initial stressors may not only persist but also contribute to the development of additional stressors [12]. As adolescent stressors can escalate over the life course, continuing beyond adolescence, and exacerbating mental health problems when not dealt with appropriately, by understanding the contributors to adolescent stress such as the levels and major causes (i.e., stressors), we will be better equipped to improve their mental health.

According to Compas [13], adolescent stressors can be classified into major life changes, chronically stressful conditions, and day-to-day hassles. Adolescents most frequently face day-to-day hassles, such as problems with family or peer relationships and academic difficulties [14]. More than about one-fourth of adolescents report experiencing serious stressors such as the death of significant others, and chronic stressors, all of which significantly impair their well-being [15]. In addition, social experiences and relational problems are the most common and significant stressors reported by adolescents [15]. However, the stress level and specific kinds of stressors to which each adolescent is susceptible can differ as a result of sociodemographic characteristics such as age, cultural background, gender, and economic status [3,9,16]. For instance, girls tend to experience greater levels of stress than do boys in all stress domains [8,16], and ethnic minority students (i.e., black students) report higher stress than do students from the ethnic majority [17]. Individuals from poor families are more likely to experience stressors regarding family circumstances, such as poor income, unfavorable neighborhood environments (e.g., residing in areas with higher crime rates) and parental discord. These disadvantageous conditions expose them to more negative life experiences that are chronic and uncontrollable [18]. As a result, individuals of lower socioeconomic status (SES) reveal poorer psychological or physical health outcomes, which may, in turn, serve as another major burden in a vicious cycle.

Most studies pertaining to Korean adolescents’ stress have concentrated on its ramifications for psychosocial adaptation. For instance, stress has been researched as a risk factor for or mediator in suicidal ideation, school violence, and poor academic performance [19,20,21,22]; or is studied in relation to adverse health/behavioral habits including problematic Internet use, drinking and smoking [23,24]. However, there has been no study on the current stress levels and stressor types among Korean adolescents, which could be helpful for the development and implementation of effective stress management. Therefore, we sought to examine both the stress levels and major stressors of Korean adolescents, and to determine how these differ by gender, educational level, place of residence, and SES using recent Korean national survey data.

## 2. Materials and Methods

### 2.1. Participants

Data from the 2015 Korea Youth Risk Behavior Web-based Survey (KYRBS), an online self-report survey aimed at identifying the health behaviors of Korean adolescents attending middle school (aged between 12 and 15) and high school (aged between 15 and 18) was used in this study [25]. The KYRBS is a government-approved national survey based on the National Health Promotion Act, which has been conducted annually since 2005 by the Korea Centers for Disease Control and Prevention in an attempt to acquire the basic data necessary for planning and assessing health promotion projects. In order to maximize the representativeness of the sample, schools and classes were selected considering their location (e.g., large cities, small cities, or towns) and types (e.g., middle schools, general high schools, or specialized high schools such as those providing training for industrial professions). As a result, a total of 70,362 students from 400 middle and 400 high schools were selected as subjects of investigation.

In June 2015, the survey was conducted in each school’s computer lab under the guidance of teachers. Each student was given a brief explanation of the objectives of the study and instructions for the survey via video clip or PowerPoint presentation as well as written guidelines. Computers were randomly assigned to all students, who then accessed the online survey homepage with the participation number provided in the written guidelines. The entire survey process took 40–45 min and the students were given small gifts in return for participation. Students who were absent or took early leave from school were not included in the survey. As a result, data from 797 schools and 68,043 respondents who actually participated were used in the analysis (response rate: 96.7%) [25]. The KYRBS was approved by the Institutional Review Board of the Korea Centers for Disease Control and Prevention (2014-06EXP-02-P-A). 

### 2.2. Measures

Perceived stress was assessed with the question, “In general, how much stress do you usually feel?” Participants were asked to answer this question on a five-point Likert scale (1 = no stress, 2 = not much, 3 = a little, 4 = a lot, 5 = a great deal). Other than those who answered “no stress” to this question (*n* = 2558), 65,485 participants were then required to choose one among the seven alternatives as the primary cause of their perceived stress (i.e., a major stressor): conflicts with parents, family circumstances, conflicts with teachers, peer relational problems, academic performance, health problems, and physical appearance.

Participants’ gender, current educational level (middle or high school), and residential area (urban or rural) were also asked. Perceived level of families’ SES was obtained based on a five-point Likert scale (1 = high, 2 = above average, 3 = average, 4 = below average, 5 = low) and further divided into two categories—below average (4–5) vs. average and above (1–3)—in order to carry out binominal logistic regression analysis (Acknowledging that this categorization could be arbitrary, we present the additional results derived from the categorization of SES level as average and below (3–5) vs. above average (1–2)). 

### 2.3. Statistical Analysis

Using independent *t*-tests, we compared perceived stress levels according to gender, current educational level, residential area, and SES level. Participants’ SES, which had originally been measured on a five-point Likert scale, was divided into two categories (i.e., average and below vs. above average) so that *t*-tests and logistic regression analyses could be made possible in accordance with other bivariate variables. In consideration of multiple comparisons, the significance level for each *t*-test was adjusted to <0.0125 (0.05/4) using the Bonferroni adjustment to reduce Type I errors.

The independent associations of gender, current educational level, residential area, and SES on the perceived major stressors were then examined using logistic regression analyses. Adjusted odds ratios (AORs) and 95% confidence intervals (CIs) were calculated for each independent variable, with the influence of other variables excluded. The reference groups for AORs were: boys for gender, middle school for current educational level, urban for residential area, and higher SES for SES. In this case, the significance level for each analysis was also adjusted to <0.0018 (0.05/7 × 4) by the Bonferroni method to reduce Type I errors.

## 3. Results

Respondents’ mean age was 14.96 ± 1.74 years, with a range of 12 to 18 years. Of the 68,043 respondents, 51.74% (35,204) were male and 50.71% (34,299) were middle school students. In addition, 91.69% (62,388) were urban residents and 83.02% (56,492) reported average or higher SES. Differences in stress levels according to gender, educational level, residential area, and SES level are shown in Table 1. Independent *t*-tests revealed that perceived stress levels were higher in girls than in boys (*t* = 41.54, *p* < 0.001) and in high school students than in middle school students (*t* = 28.53, *p* < 0.001), but were comparable between urban and rural residents (*t* = 0.01, *p* = 0.994). In addition, stress levels were higher in adolescents of lower SES (*t* = 31.89, *p* < 0.001) (The result of the *t*-test when the SES level was divided into above average (1–2) vs. average and below (3–5) was as follows: *t* = 24.57, *p* < 0.001. Means and standard deviations were 3.07 (0.98) for the above average group and 3.26 (0.92) for the average and below group). 

Distribution of major stressors among Korean adolescents according to gender, educational level, residential area, and SES level is shown in Table 2.

After adjusting for gender, residential area, and SES level, high school students had greater odds of reporting problems related to family circumstances (AOR = 1.28; 95% CI = 1.24–1.32) and academic performance (AOR = 1.77; 95% CI = 1.72–1.83) as major stressors compared to middle school students. However, high school students had lower odds of reporting conflicts with parents (AOR = 0.49; 95% CI = 0.46–0.51), conflicts with teachers (AOR = 0.82; 95% CI = 0.74–0.91), peer relational problems (AOR = 0.78; 95% CI = 0.74–0.83), and physical appearance (AOR = 0.73; 95% CI = 0.69–0.77).

When adjusting for gender, current educational stage, and SES level, rural residents had greater odds of reporting conflicts with teachers (AOR = 1.42; 95% CI = 1.21–1.67), peer relational problems (AOR = 1.37; 95% CI = 1.26–1.50), and health problems (AOR = 1.55; 95% CI = 1.34–1.79) as major stressors compared to urban residents. However, rural residents were less likely to report conflicts with parents (AOR = 0.84; 95% CI = 0.77–0.92) and problems with academic performance (AOR = 0.81; 95% CI = 0.76–0.86).

Finally, after adjusting for gender, educational level and residential area, adolescents of lower SES had greater odds of reporting conflicts with parents (AOR = 1.19; 95% CI = 1.11–1.26), problems in family circumstances (AOR = 1.75; 95% CI = 1.68–1.83), peer relationships (AOR = 1.26; 95% CI = 1.17–1.35), and physical appearance (AOR = 1.26; 95% CI = 1.18–1.34). However, students with lower SES levels had lower odds of reporting stress related to academic performance (AOR = 0.48, 95% CI = 0.46–0.50) (Table 3). (When SES level was divided into above average (1–2) vs. average and below (3–5), the results of the logistic regression analyses were as follows: adolescents with lower SES had greater odds of reporting conflicts with parents (AOR = 1.08; 95% CI = 1.03–1.13, *p* < 0.05), problems in family circumstances (AOR = 1.21; 95% CI = 1.17–1.25, *p* < 0.001), conflicts with teachers (AOR = 0.87; 95% CI = 0.78–0.96, *p* < 0.05), peer relational problems (AOR = 1.17; 95% CI = 1.10–1.23, *p* < 0.001), and physical appearance (AOR = 1.15; 95% CI = 1.10–1.21, *p* < 0.001). However, students with lower SES had lower odds of reporting stress related to academic performance (AOR = 0.74; 95% CI = 0.71–0.76, *p* < 0.001).)

## 4. Discussion

In this study, stress levels, as well as the extent to which certain types of stressors were related to specific groups of Korean adolescents were identified using data from the 2015 KYRBS. The results of this study are as follows. Firstly, in general, higher levels of stress were related to females, high school students, as well as lower SES. These results are important in that they identify adolescent subgroups that are especially vulnerable to stress. In this way, people in charge of adolescents’ stress management (e.g., teachers or school counsellors) can stay focused on and thus quickly respond to the needs of potential service users.

Secondly, adolescents’ major stressors varied by gender, educational level, residential area, and SES level, emphasizing the importance of considering individual characteristics when understanding adolescents’ stress. Specifically, girls were more likely to cite relationship- and appraisal-related factors (e.g., peer relationship problems and physical appearance) as major stressors. Adolescent girls might find peer relationship problems more stressful because they tend to care more about their close friendships than do boys [26]. Furthermore, physical appearance might be a greater stressor for girls because of their generally higher rates of body dissatisfaction [27], which is significantly associated with greater emotional distress and depressive symptoms [28,29]. Conversely, for boys, health- and conflict-related factors were more likely to be reported as major stressors. Conflicts with teachers might be a greater stressor in part because boys tend to receive more negative attention from teachers compared to girls [30,31]. In addition, girls tended to report higher stress regarding academic performance whereas boys reported health problems as one of their major stressors. A possible explanation for this discrepancy could be that scholastic competence is related to positive self-worth for girls, whereas perceived bodily and athletic competences are more important among boys [32].

As for the results for educational level, high school students were more likely to report family circumstances and academic performance as major stressors than were middle school students. This possibly relates to adolescent identity development, which is characterized by a series of crises and decisions in numerous domains of life concerning one’s interpersonal relationships, ideology, and future education or occupation [33,34]. Adolescents’ exploration of these major identity issues tends to increase with age [35,36]. Moreover, high school students might be more stressed about their academic performance because their grades largely determine which university they get into. In South Korea, going to a high-ranking university is a major concern for students because it can help them secure a good job, high social status, and a good marriage [37]. Middle school students, however, were more likely to report conflict-related factors, including conflicts with parents or teachers, as major stressors. This result accords with prior studies showing that perceived conflicts with parents peak in early and middle adolescence, and thereafter decrease [38,39].

Urban adolescents were more likely to report academic performance and conflicts with parents as major stressors than were rural adolescents. Urban adolescents might experience greater stress from academic performance because they tend to aspire to attend university, whereas rural students more often seek out work or enter a trade after high school [40]. The greater stress from conflicts with parents in urban adolescents may, in part, be because their parents show higher involvement in various life domains including academic achievement [41]. For rural adolescents, conflicts with teachers and peer relationship problems were more likely to be reported as major stressors. This may be because conflicts have a greater impact on adolescents’ stress levels in closed settings, where future exchanges with the same individuals are inevitable, compared to in open-field settings, where alternative relationships exist [42]. As rural communities tend to be closed settings, interpersonal conflicts with peers and teachers might be stronger stressors for rural adolescents [43].

Adolescents of lower SES were more likely to experience higher stress from various domains including disadvantageous family conditions, relational problems, as well as higher apprehension related to physical appearance. This result is consistent with previous studies in which lower SES levels tend to expose children and adolescents more frequently to various negative stressful events including severe conflicts or dissolution of families, frequent moves, as well as unfavorable circumstances for cognitive and academic attainment [44]. In contrast, higher SES was solely associated with greater stress in academic performance. This result indicates that the struggle for higher academic achievement, which is explicitly or implicitly demanded from their advantageous environmental conditions, is a primary and unique stressor for adolescents of higher SES, though multiple studies repeatedly confirm that academic performance itself is significantly low in adolescents in poorer conditions [44].

This study had several limitations regarding methodology. First and foremost, this study utilized only self-reported data. SES level in particular, is likely to be biased when self-reported as people may be reluctant to identify themselves as low SES in order to keep up appearances. Nevertheless, the fact that the survey was conducted anonymously online, making each response unidentifiable, could have mitigated the potential biases. In addition, the item for SES was not about the parents’ occupation or income, to which participants might not be able to identify correctly [45], or feel more uncomfortable and be tempted to falsely report; instead it asked more broadly about how affluent the student perceived their family to be, which participants were expected to feel more comfortable about answering. In addition, measurement of perceived level of stress was based solely on the result of a single self-reported item, usually more prone to psychometric flaws. However, there has been some evidence that single-item measures of stress could be comparable to multi-item instruments. Specifically, single-item measures have been suggested as having good test-retest reliability and validity, showing strong associations with other validated measures of psychological as well as psychosocial symptoms and well-being [46,47]. Therefore, single-item instruments could be more practical than multiple-item measures for the assessment of stress in large sample studies.

## 5. Conclusions

In conclusion, adolescents’ stress levels and primary stressors are characteristically associated with individual factors such as gender, current educational stage, place of residence, and family SES. Government officials and teachers should consider these factors and try to implement stress management approaches suited to individual demands when attempting to alleviate adolescents’ stress. For instance, prospective middle school students could be offered psychoeducation on interpersonal changes or potential conflicts and how to deal with such problems in a healthier way. In addition, given that the majority of students in Korea reside in urban areas and suffer from stress regarding academic performance, Korean adolescents would benefit most if preventative interventions on relieving academic stress are developed and implemented.

## Figures and Tables

**Table 1:** Differences in stress levels among Korean adolescents according to individual characteristics.

Characteristics	Mean (SD)	*t*	*p*-Value
Gender		41.54	<0.001
Boys (*n* = 35,204)	3.05 (0.97)		
Girls (*n* = 32,839)	3.35 (0.90)		
Educational level		28.53	<0.001
Middle school (*n* = 34,299)	3.09 (0.96)		
High school (*n* = 33,336)	3.30 (0.92)		
Residential area		0.01	0.994
Urban (*n* = 62,388)	3.19 (0.95)		
Rural (*n* = 5655)	3.19 (0.96)		
Socioeconomic status		31.89	<0.001
Higher SES (1–3) (*n* = 56,492)	3.14 (0.94)		
Lower SES (4–5) (*n* = 11,551)	3.45 (0.95)		

**Table 2:** Distribution of major stressors among Korean adolescents according to gender, educational level, residential area, and socioeconomic status level.

Stressors	Boys (*n* = 33,254)	Girls (*n* = 32,231)	Middle School (*n* = 32,640)	High School (*n* = 32,845)	Urban (*n* = 60,053)	Rural (*n* = 5432)	Higher SES (*n* = 54,203)	Lower SES (*n* = 11,282)
	*n* (%)	*n* (%)	*n* (%)	*n* (%)	*n* (%)	*n* (%)	*n* (%)	*n* (%)
Conflicts with parents	4906 (14.8)	3274 (10.2)	5302 (16.2)	2878 (8.8)	7584 (12.6)	596 (11.0)	6727 (12.4)	1453 (12.9)
Family circumstances	1220 (3.7)	1086 (3.4)	1002 (3.1)	1304 (4.0)	2064 (3.4)	242 (4.5)	18,500 (32.7)	5458 (47.3)
Conflicts with teachers	1089 (3.3)	465 (1.4)	849 (2.6)	705 (2.1)	1378 (2.3)	176 (3.2)	1311 (2.4)	243 (2.2)
Peer relational problems	2539 (7.6)	3308 (10.3)	3201 (9.8)	2646 (8.1)	5221 (8.7)	626 (11.5)	4686 (8.6)	1161 (10.3)
Academic performance	19,123 (57.5)	19,576 (60.7)	17,360 (53.2)	21,339 (65.0)	35,769 (59.6)	2930 (53.9)	33,475 (61.8)	5224 (46.3)
Health problems	1217 (3.7)	520 (1.6)	893 (2.7)	844 (2.6)	1524 (2.5)	213 (3.9)	1422 (2.6)	315 (2.8)
Physical appearance	3160 (9.5)	4002 (12.4)	4033 (12.4)	3129 (9.5)	6513 (10.8)	649 (11.9)	5755 (10.6)	1407 (12.5)

**Table 3:** Differences in major stressors among Korean adolescents according to gender, educational level, residential area, and SES level.

Stressors	Gender ^a^	Educational Level ^b^	Residential Area ^c^	SES Level ^d^
Conflicts with parents	0.65 (0.62–0.68) **	0.49 (0.46–0.51) **	0.84 (0.77–0.92) **	1.19 (1.11–1.26) **
Family circumstances	1.65 (1.60–1.71) **	1.28 (1.24–1.32) **	1.03 (0.98–1.10)	1.75 (1.68–1.83) **
Conflicts with teachers	0.43 (0.39–0.49) **	0.82 (0.74–0.91) **	1.42 (1.21–1.67) **	0.93 (0.81–1.07)
Peer relational problems	1.39 (1.32–1.47) **	0.78 (0.74–0.83) **	1.37 (1.26–1.50) **	1.26 (1.17–1.35) **
Academic performance	1.15 (1.11–1.19) **	1.77 (1.72–1.83) **	0.81 (0.76–0.86) **	0.48 (0.46–0.50) **
Health problems	0.43 (0.39–0.48) **	0.93 (0.85–1.03)	1.55 (1.34–1.79) **	1.08 (0.96–1.23)
Physical appearance	1.36 (1.29–1.42) **	0.73 (0.69–0.77) **	1.11 (1.02–1.21) *	1.26 (1.18–1.34) **

In logistic regression analysis, boys, middle school, urban area, and higher SES were treated as the reference. ^a^, odds ratios adjusted for educational level, residential area, and SES level; ^b^, odds ratios adjusted for gender, residential area, and SES level; ^c^, odds ratios adjusted for gender, educational level, and residential area; ^d^, odds ratios adjusted for gender, educational level, and residential area. * *p* < 0.05, ** *p* < 0.001.

## References

[1] Byrne D.G., Davenport S., Mazanov J. (2007). Profiles of adolescent stress: The development of the adolescent stress questionnaire (ASQ). J. Adolesc..

[2] Casey B.J., Jones R.M., Levita L., Libby V., Pattwell S.S., Ruberry E.J., Soliman F., Somerville L.H. (2010). The storm and stress of adolescence: Insights from human imaging and mouse genetics. Dev. Psychobiol..

[3] Cicchetti D., Rogosch F.A. (2002). A developmental psychopathology perspective on adolescence. J. Consult. Clin. Psychol..

[4] Williams K., McGillicuddy-De Lisi A. (1999). Coping strategies in adolescents. J. Appl. Dev. Psychol..

[5] Miller D.B., Townsend A. (2005). Urban hassles as chronic stressors and adolescent mental health: The urban hassles index. Brief Treat. Crisis Interv..

[6] Wadsworth M.E., Compas B.E. (2002). Coping with family conflict and economic strains: The adolescent perspective. Soc. Res. Adolesc..

[7] Hjern A., Alfven G., Ostberg V. (2008). School stressors, psychological complaints and psychosomatic pain. Acta Paediatr..

[8] Moksnes U.K., Moljord I.E.O., Espnes G.A., Bryne D.G. (2010). The association between stress and emotional states in adolescents. Pers. Individ. Differ..

[9] Hampel P., Petermann F. (2006). Perceived stress, coping, and adjustment in adolescents. J. Adolesc. Health.

[10] Turner R.J., Lloyd D.A. (1995). Lifetime traumas and mental health: The significance of cumulative adversity. J. Health Soc. Behav..

[11] Adams D.M., Overholser J.C., Spirito A. (1994). Stressful life events associated with adolescent suicide attempts. Can. J. Psychiatry Rev. Can. Psychiatr..

[12] Pearlin L.I., Schieman S., Fazio E.M., Meersman S.C. (2005). Stress, health, and the life course: Some conceptual perspectives. J. Health Soc. Behav..

[13] Compas B.E. (1987). Coping with stress during childhood and adolescence. Psychol. Bull..

[14] Goldberg E.L., Comstock G.W. (1980). Epidemiology of life events: Frequency in general populations. Am. J. Epidemiol..

[15] Zimmer-Gembeck M.J., Skinner E.A. (2008). Adolescents’ coping with stress: Development and diversity. Prev. Res..

[16] Ge X., Lorenz F.O., Conger R.D., Elder G.H., Ronald L. (1994). Trajectories of stressful life events and depressive symptoms during adolescence. Dev. Psychol..

[17] Goodman E., McEwen B.S., Dolan L.M., Schafer-Kalkhoff T., Adler N.E. (2005). Social disadvantage and adolescent stress. J. Adolesc. Health Off. Publ. Soc. Adolesc. Med..

[18] Landis D., Gaylord-Harden N.K., Malinowski S.L., Grant K.E., Carleton R.A., Ford R.E. (2007). Urban adolescent stress and hopelessness. J. Adolesc..

[19] Park S., Lee Y., Jang H., Jo M. (2017). Violence victimization in korean adolescents: Risk factors and psychological problems. Int. J. Environ. Res. Public Health.

[20] Park S., Lee Y. (2016). Factors that affect suicide attempts of adolescents in multicultural families in Korea. Int. J. Environ. Res. Public Health.

[21] Kim Y.J., Moon S.S., Lee J.H., Kim J.K. (2018). Risk factors and mediators of suicidal ideation among korean adolescents. Crisis.

[22] Lee S., So W.Y., Sung D.J. (2015). Association between chronic mental stress and academic performance among korean adolescents. Univ. Psychol..

[23] Park S. (2014). Associations of physical activity with sleep satisfaction, perceived stress, and problematic internet use in korean adolescents. BMC Public Health.

[24] Kim S. (2010). The effects of psychosocial predictors during middle school and high school on adolescent drinking and smoking. J. Soc. Sci..

[25] Disease Control and Prevention (2015). The Eleventh Korea Youth Risk Behavior Web-Based Survey.

[26] Benenson J.F., Benarroch D. (1998). Gender differences in responses to friends’ hypothetical greater success. J. Early Adolesc..

[27] Thompson J.K., Heinberg L.J., Altabe M., Tantleff-Dunn S. (1999). Exacting Beauty: Theory, Assessment, and Treatment of Body Image Disturbance.

[28] Ohring R., Graber J.A., Brooks-Gunn J. (2002). Girls’ recurrent and concurrent body dissatisfaction: Correlates and consequences over 8 years. Int. J. Eat. Disord..

[29] Allgood-Merten B., Lewinsohn P.M., Hops H. (1990). Sex differences and adolescent depression. J. Abnorm. Psychol..

[30] Beaman R., Wheldall K., Kemp C. (2006). Differential teacher attention to boys and girls in the classroom. Educ. Rev..

[31] Younger M., Warrington M., Williams J. (1999). The gender gap and classroom interactions: Reality and rhetoric?. Br. J. Sociol. Educ..

[32] Piek J.P., Baynam G.B., Barrett N.C. (2006). The relationship between fine and gross motor ability, self-perceptions and self-worth in children and adolescents. Hum. Mov. Sci..

[33] Erikson E. (1968). Youth: Identity and Crisis.

[34] Vondracek F.W. (1992). The construct of identity and its use in career theory and research. Career Dev. Q..

[35] Bosma H. (1985). Identity Development in Adolescence. Coping with Commitments. Ph.D. Thesis.

[36] Markstrom-Adams C., Adams G.R. (1995). Gender, ethnic group, and grade differences in psychosocial functioning during middle adolescence?. J. Youth Adolesc..

[37] Bae J., Lee M. (1988). Substance of Korean Education: How Korean People Think Education.

[38] Furman W., Buhrmester D. (1992). Age and sex differences in perceptions of networks of personal relationships. Child Dev..

[39] Allison B.N., Schultz J.B. (2004). Parent-adolescent conflict in early adolescence. Adolescence.

[40] Gándara P., Gutiáez D., O’Hara S. (2001). Planning for the future in rural and urban high schools. J. Educ. Stud. Placed Risk.

[41] Jeynes W.H. (2007). The relationship between parental involvement and urban secondary school student academic achievement: A meta-analysis. Urban Educ..

[42] Laursen B., Collins W.A. (1994). Interpersonal conflict during adolescence. Psychol. Bull..

[43] Elgar F.J., Arlett C., Groves R. (2003). Stress, coping, and behavioural problems among rural and urban adolescents. J. Adolesc..

[44] Bradley R.H., Corwyn R.F. (2002). Socioeconomic status and child development. Annu. Rev. Psychol..

[45] Currie C.E., Elton R.A., Todd J., Platt S. (1997). Indicators of socioeconomic status for adolescents: The WHO health behaviour in school-aged children survey. Health Educ. Res..

[46] Elo A.-L., Leppänen A., Jahkola A. (2003). Validity of a single-item measure of stress symptoms. Scand. J. Work. Environ. Health.

[47] Littman A.J., White E., Satia J.A., Bowen D.J., Kristal A.R. (2006). Reliability and validity of 2 single-item measures of psychosocial stress. Epidemiology.

